# Assignment of the Internal Vibrational Modes of C_70_ by Inelastic Neutron Scattering Spectroscopy and Periodic-DFT

**DOI:** 10.1002/open.201500069

**Published:** 2015-05-20

**Authors:** Keith Refson, Stewart F Parker

**Affiliations:** [a] ISIS Facility, Science and Technology Facilities Council (STFC), Rutherford Appleton LaboratoryChilton, Didcot, OX11 0QX, UK; [b] Department of Physics, Royal Holloway, University of LondonEgham, TW20 0EX, UK

**Keywords:** Ab initio calculations, C_70_, fullerenes, inelastic neutron scattering spectroscopy, infrared spectroscopy, Raman spectroscopy

## Abstract

The fullerene C_70_ may be considered as the shortest possible nanotube capped by a hemisphere of C_60_ at each end. Vibrational spectroscopy is a key tool in characterising fullerenes, and C_70_ has been studied several times and spectral assignments proposed. Unfortunately, many of the modes are either forbidden or have very low infrared or Raman intensity, even if allowed. Inelastic neutron scattering (INS) spectroscopy is not subject to selection rules, and all the modes are allowed. We have obtained a new INS spectrum from a large sample recorded at the highest resolution available. An advantage of INS spectroscopy is that it is straightforward to calculate the spectral intensity from a model. We demonstrate that all previous assignments are incorrect in at least some respects and propose a new assignment based on periodic density functional theory (DFT) that successfully reproduces the INS, infrared, and Raman spectra.

## Introduction

Fullerene science may be said to have been born in 1990, when the route to macroscopic quantities of the materials was invented.^1^ This prompted an intense and continuing effort, both experimental and theoretical, to understand and exploit the properties of these novel forms of carbon. Areas of interest span physics,^2^ chemistry,^3^ biology,^4^ and astronomy.^5^,^6^ Most of the activity has focussed on C_60_ because of the iconic nature of the icosohedral symmetry and for the practical reason that it is the most readily available of the fullerenes.

**Figure 1:**
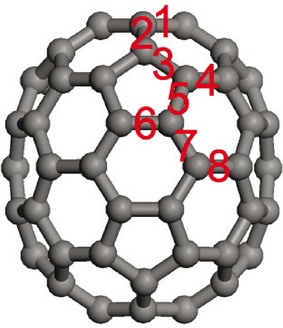
View of C_70_ showing the definitions of the bonds.

The second most abundant fullerene is C_70_ (Figure 1). This may be considered as the shortest possible nanotube capped by a hemisphere of C_60_ at each end. The additional ten carbon atoms have a profound effect on the molecule. The idealised (gas-phase) symmetry is reduced to *D*_5*h*_ from *I*_h_. This means that there are now eight distinct types of C−C bond rather than the two found in C_60_. The number of internal vibrational modes increases to 204 from 174. These are classified as:

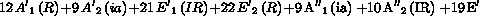
1

Thus, there are many more modes in C_70_ than C_60_:122 vs. 46. The reduction in symmetry does have one advantage—in C_70_, 84 modes (31 infrared and 53 Raman) are potentially observable by infrared and Raman spectroscopies, whereas only 14 (4 infrared and 10 Raman) are allowed in C_60_.

In the solid state, the consequences of the additional carbon atoms are equally striking. From the sublimation temperature to 260 K, C_60_ undergoes rapid rotation and the structure can be described as a face-centred cubic (fcc) lattice of spheres, space group Fm
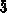
m with *Z*=1 in the primitive cell.^7^ Below 260 K, an orientationally ordered simple cubic phase is obtained, space group Pa
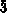
with *Z*=4 in the primitive cell.^8^ In contrast, at room temperature, the phase obtained with C_70_ may be fcc or hexagonal close packed (hcp); these may partially order as the rotation about the short axis and then the long axis is quenched on cooling. The low temperature phase has been proposed to be orthorhombic, *Pbnm*,^9^ with *Z*=4 and also as monoclinic.^10^ Whether the room temperature phase is fcc or hcp, whether there are one or two rotator phases, and whether the low temperature phase is orthorhombic or monoclinic depend sensitively on sample history, cooling rate, and purity, particularly the amount of C_60_ present. The complex crystallography of C_70_ is well summarised elsewhere.^11^

The assignment of the internal modes of C_70_ is largely based on solid-state spectra; the only gas-phase data^12^ identified eight infrared active fundamentals. The infrared and Raman spectra have been measured several times;^13^–^20^ as with C_60_ it was realised that all the modes are allowed in inelastic neutron scattering (INS) spectroscopy,^21^ and a low-quality spectrum has been reported.^22^ Most^19^,^23^–^25^ computational studies assume *D*_5*h*_ symmetry, that is, they use the isolated molecule approximation. One study^26^ employed Car–Parrinello molecular dynamics so as to include solid-state effects. A critical test of the reliability of a calculation is how well it reproduces the INS spectrum. We have previously adopted this approach to completely assign the vibrational spectrum of C_60_ and have shown that all previous assignments were incorrect in some respect.^27^ Two papers using different computational methods have done so for C_70_; both report good agreement.^23^,^25^ However, the quality of the spectrum, particularly in the 800–1600 cm^−1^ region is insufficient to make this a useful comparison. In this work, we report a new INS spectrum obtained from a large sample (9 g, about 18 times larger than that previously used) recorded at the highest resolution available, combined with a periodic density functional theory (DFT) calculation that includes solid-state effects. Together, these allow a stringent test of the published assignments and a new assignment of all of the internal modes of C_70_.

## Results and Discussion

**Figure 2:**
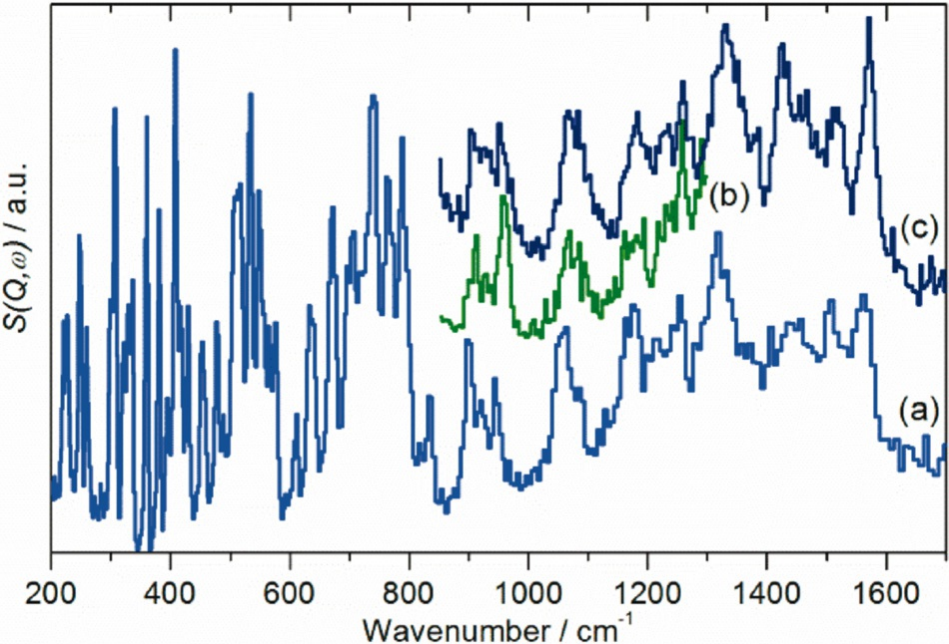
Vibrational spectra of C_70_ at 7 K. a) INS spectrum recorded on TOSCA, b) INS spectrum recorded on MAPS with an incident energy of 1452 cm^−1^, and c) INS spectrum recorded on MAPS with an incident energy of 2017 cm^−1^.

**Figure 3:**
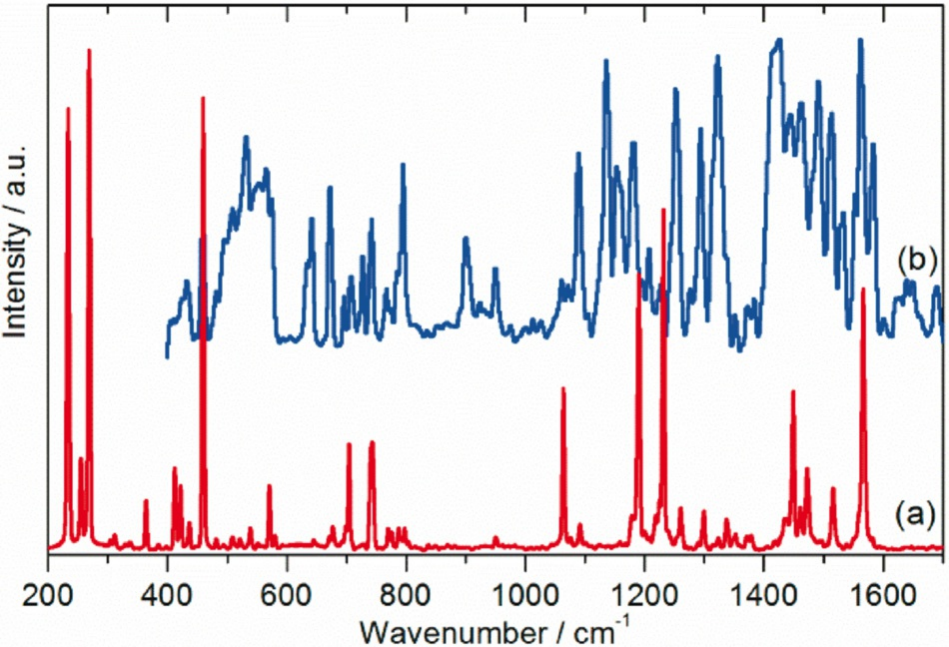
a) Raman spectrum at 7 K after correction for the instrument response and sloping baseline. This was recorded simultaneously with the TOSCA spectrum (Figure 1 a). b) Infrared spectrum recorded at 113 K using ATR after baseline correction.

As described in the Introduction, there is no agreement as to the exact form of the low-temperature structure of C_70_, with both orthorhombic and monoclinic structures proposed.^9^–^11^ Since fivefold symmetry is incompatible with long-range order,^28^ it follows that the site symmetry must be simultaneously a subgroup of *D*_5*h*_ and also a permitted site symmetry of *Pbnm* or a monoclinic space group. Only *C*_s_, *C*_2_, and *C*_1_ meet these constraints. Spectroscopically, all three are equivalent: all degeneracies are lifted and all modes are allowed in both the infrared and Raman spectra. (The three possibilities are only distinguishable by single crystal studies with polarised radiation.) However, the intensities will vary considerably, and modes that are forbidden under *D*_5*h*_ symmetry are likely to be weak in the solid-state spectra and may be confused with combination modes, as found with C_60_^29^,^30^ All the modes are allowed in INS spectroscopy and all will have similar intensities, since all the atoms in the mode have the same scattering cross section and will have similar amplitudes of vibration. Figure 2 shows the INS spectra recorded with the TOSCA and MAPS instruments (ISIS Facility, Rutherford Appleton Laboratory, Chilton, UK). Figure 3 a shows the Raman spectrum at 20 K recorded simultaneously with the TOSCA spectrum, while Figure 3 b shows the infrared spectrum at 113 K recorded by attenuated total reflectance (ATR). In comparison to previous work,^22^ the INS spectra exhibit a signal-to-noise ratio more than an order of magnitude better and with significantly improved resolution, particularly in the 800–1600 cm^−1^ region. While the infrared and Raman spectra show many modes across the range 200–3000 cm^−1^, there is a sharp cut-off at 1600 cm^−1^ in the INS spectra, demonstrating that all modes to higher energy must be overtones or combinations.

**Figure 4:**
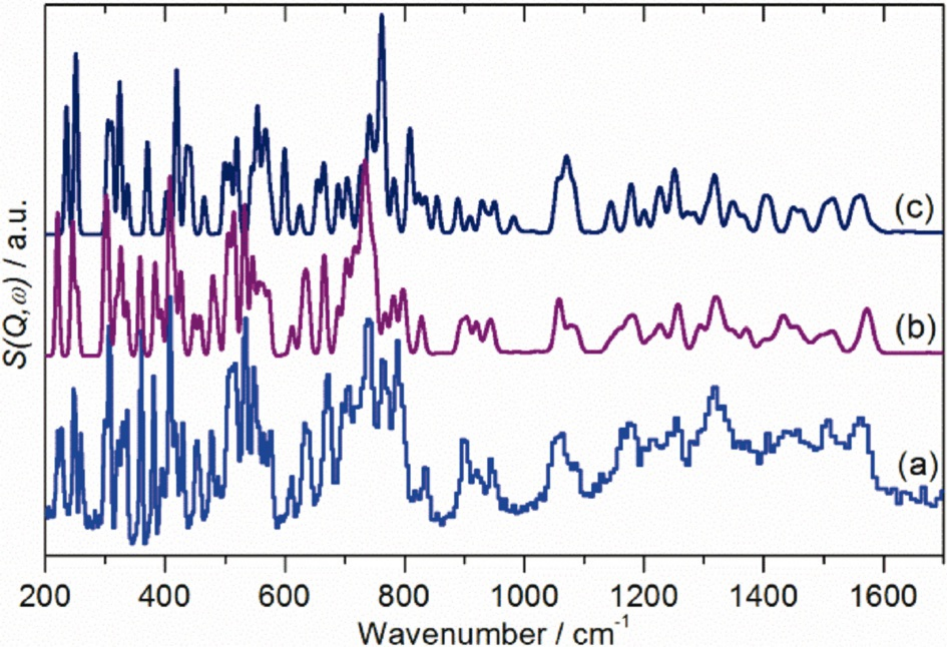
Comparison of the a) TOSCA INS spectrum of C_70_ at 7 K with spectra simulated using literature assignments: b) for the gas phase by GAUSSIAN 98 (*D*_5*h*_ symmetry)^19^ and c) by an ab initio molecular dynamics simulation.^26^

The need for a new assignment is demonstrated in Figure 4, which compares our new INS spectrum (Figure 4 a) with the INS spectra predicted by literature results assuming an isolated (gas phase) molecule^19^ (Figure 4 b) and by an ab initio molecular dynamics simulation^26^ (Figure 4 c). It can be seen that while the overall profile is approximately correct, both simulated spectra are wrong in detail, particularly in the 450–800 cm^−1^ region. For the present work, we have chosen to model the system with ab initio lattice dynamics (as implemented in the DFT code, CASTEP), that was successfully used for C_60_.^27^

The only structure that has the atomic coordinates available is the *Pbnm*^9^ structure with *Z*=4 and *C*_s_ site symmetry, where the horizontal mirror plane of the *D*_5*h*_ point group is coincident with the crystallographic mirror plane of *Pbnm*. We chose to simplify the problem by removing three molecules from the unit cell. This reduces the space-group symmetry to *Pm* but retains the point-group symmetry reduction that must be present in the solid state.

Table 1 compares the available structural data^9^,^31^,^32^ with the results of the CASTEP calculation for the *Pm* structure and that of the idealised *D*_5*h*_ gas phase molecule calculated using GAUSSIAN 03. For ease of comparison with other work, we have adopted the same definitions of the bonds as used previously (see Figure 1).^9^ It can be seen that both calculations are in good agreement with the experimental data and with each other; the differences rarely exceed ±0.1 Å. The only discrepancy is with the experimental gas phase value for bond 8; however, in comparison to the other distances in the molecule, this appears anomalous. We note that the longest experimental distance in C_60_ is 1.487 Å.^8^

**Table 1:** Comparison of observed and calculated intramolecular bond distances (Å) in C_70_.

Bond[a]	Gas phase [*D*_5*h*_]			Solid state		
	Ab initio	Expt.[b]	Ab initio *Pm*	Expt. *Pbnm*[c]	Expt.[d]
1	1.461	1.461(8)	1.453	1.434(8)	1.459(4)
2	1.405	1.388(16)	1.401	1.377(10)	1.385(7)
3	1.457	1.453(11)	1.447	1.443(9)	1.449(6)
4	1.398	1.386(25)	1.393	1.369(10)	1.376(7)
5	1.455	1.468(11)	1.446	1.442(7)	1.460(1)
6	1.446	1.425(14)	1.437	1.394(11)	1.438(4)
7	1.428	1.405(13	1.419	1.418(7)	1.417(6)
8	1.477	1.538(19)	1.466	1.457(12)	1.479(7)

[a] See Figure 1 for the definition of the bonds.

[b] Ref. 31.

[c] Ref. 9.

[d] Ref. 32.

**Figure 5:**
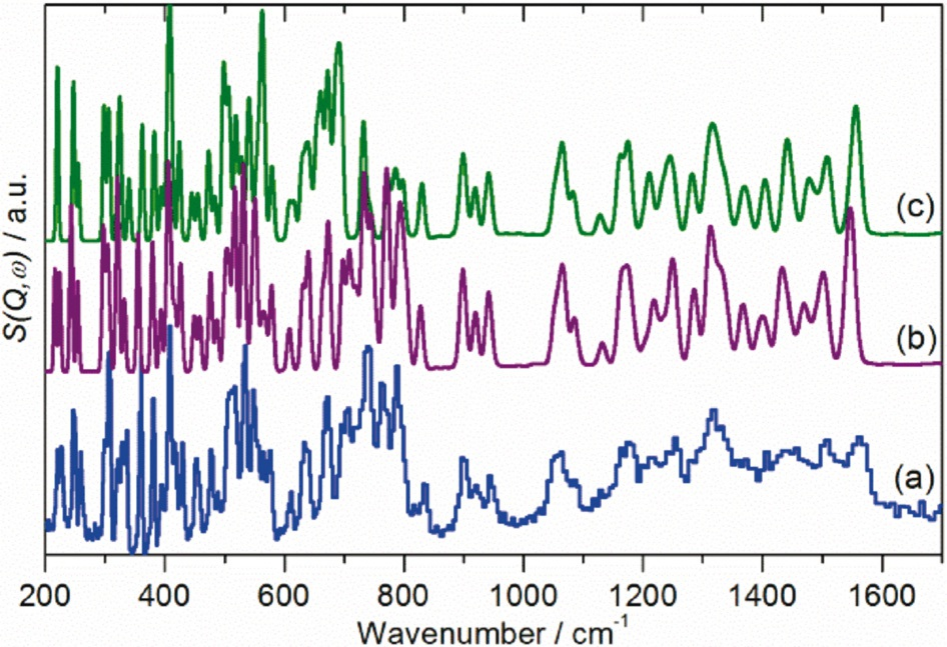
Comparison of the a) TOSCA INS spectrum of C_70_ at 7 K with that calculated for the b) solid state by CASTEP (site symmetry *C*_s_) and c) for the gas phase by GAUSSIAN 03 (*D*_5*h*_ symmetry).

A comparison of the TOSCA INS spectrum with that calculated for the gas phase and the solid state is shown in Figure 5. It can be seen from the comparisons of the observed and calculated spectra that the present results provide a better description of the vibrational modes of C_70_ than all previous computational studies.^19^,^23^–^25^ In most cases, these have imposed *D*_5*h*_ point-group symmetry on the calculation, that is, a gas-phase calculation, hence mode assignment is straightforward. In our case, we have a *C*_s_ symmetry molecule, and relating the modes back to the parent *D*_5*h*_ symmetry is more complex. To proceed, we note that the highest subgroup of *D*_5*h*_ that is compatible with translational symmetry is *C*_2*v*_. Imposing *C*_2*v*_ symmetry on the *Pm* C_70_ structure raises the crystal symmetry to *Pmm*2. This structure was then geometry optimised, and the vibrational modes were calculated. This results in an INS spectrum that has the same profile as that from the *Pm* structure, but slightly shifted to lower wavenumber. Thus, the modes have been calculated in the same order in both systems. Using the correlation given in Table S1 in the Supporting Information, and assuming that modes that are degenerate under *D*_5*h*_ symmetry will be very close (a few wavenumbers at most), it can be seen that the modes can be readily assigned as *A′*, *E′*, *A“*, and *E”*.

In order to assign the modes as to whether they are symmetric or antisymmetric with respect to the improper rotation 2 *S*_5_, (subscript 1 or 2, respectively), we note that eight infrared modes^12^ are observed in the gas phase and 24 Raman modes in solution.^18^ In both cases, *D*_5*h*_ symmetry is present. Hence, the infrared active modes must be either 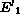
or 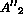, and the Raman active modes 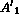, 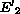, or 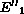; thus, these limit the possibilities for some of the modes. Additionally, inspection of Figure 5 shows that the major discrepancies between the isolated molecule (*D*_5*h*_) spectra and the solid-state (*C*_s_) spectra (observed and calculated) occur in the region 450–800 cm^−1^. Outside this region, the INS spectral patterns are remarkably similar, suggesting that the *D*_5*h*_ assignments are reliable below 450 cm^−1^ and above 800 cm^−1^. In combination, the experimental infrared and Raman transition energies with the partial gas-phase assignment enable a complete set of assignments to be made. This, for the internal modes of C_70_ with *C*_s_ symmetry in the *Pm* structure, is given in Table S2 in the Supporting Information, together with the observed INS, infrared, and Raman bands.

**Figure 6:**
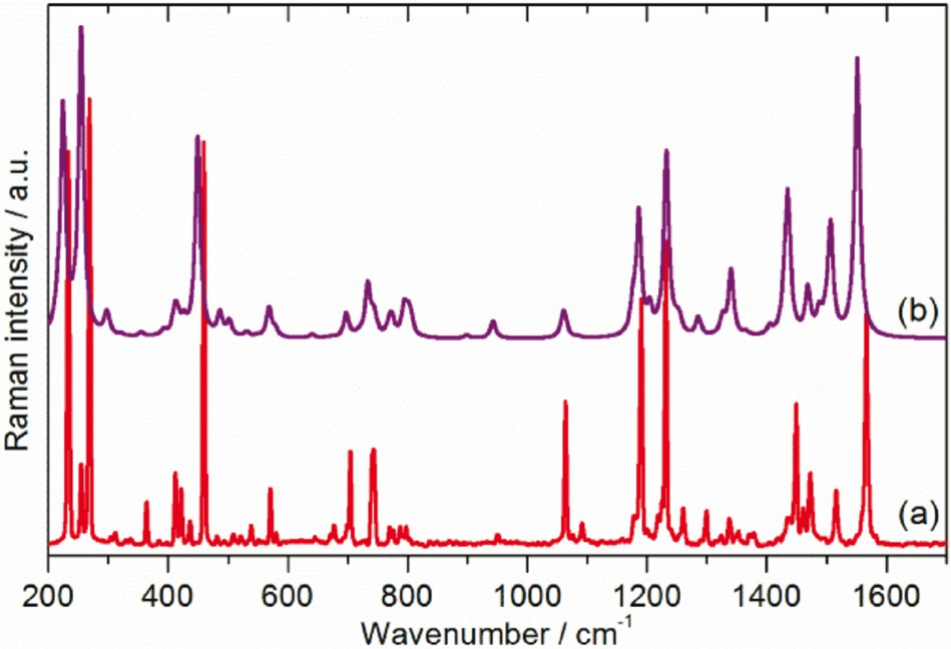
Comparison of a) the observed Raman spectrum of C_70_ at 7 K with that calculated for the solid state b) by CASTEP (site symmetry *C*_s_).

**Figure 7:**
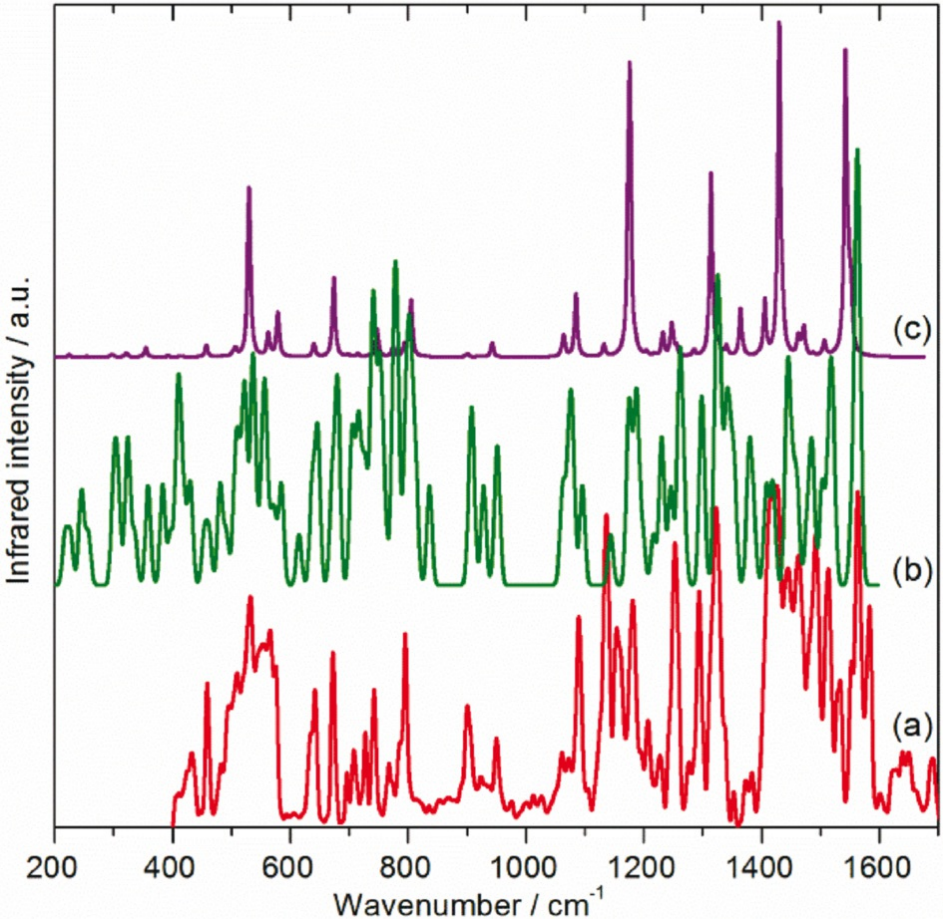
Comparison of a) the observed infrared spectrum of C_70_ at 113 K with b) the calculated VDOS and c) calculated infrared spectrum; (b) and (c) are calculated for the solid state by CASTEP with *C*_s_ site symmetry.

Further support for the validity of our model is provided by comparison of the observed and calculated Raman and infrared spectra (Figures 6 and 7). Our Raman spectrum was recorded using 785 nm excitation, which is well removed from the lowest-energy electronic absorption bands at 467 and 545 nm,^33^ so the spectrum should not be affected by resonance or preresonance effects,^18^ and the agreement is very good. The infrared spectrum (Figure 7 a), was recorded by ATR and is markedly different from those recorded by transmission infrared spectroscopy.^13^,^15^,^16^,^19^,^20^ Comparison with the vibrational density of states (VDOS) (Figure 7 b) and the calculated infrared spectrum (Figure 7 c) shows much better agreement with the former. Many more modes are observed than expected, most of which are fundamentals. It is not at all clear why this occurs. The ATR device uses a clamp to ensure good contact between the sample and the diamond ATR element; however, pressure alone cannot be the explanation, as transmission infrared measurements^20^ up to 10 GPa do not result in a similar spectrum. We (and others)^34^ have observed a similar effect in C_60_.

**Figure 8:**
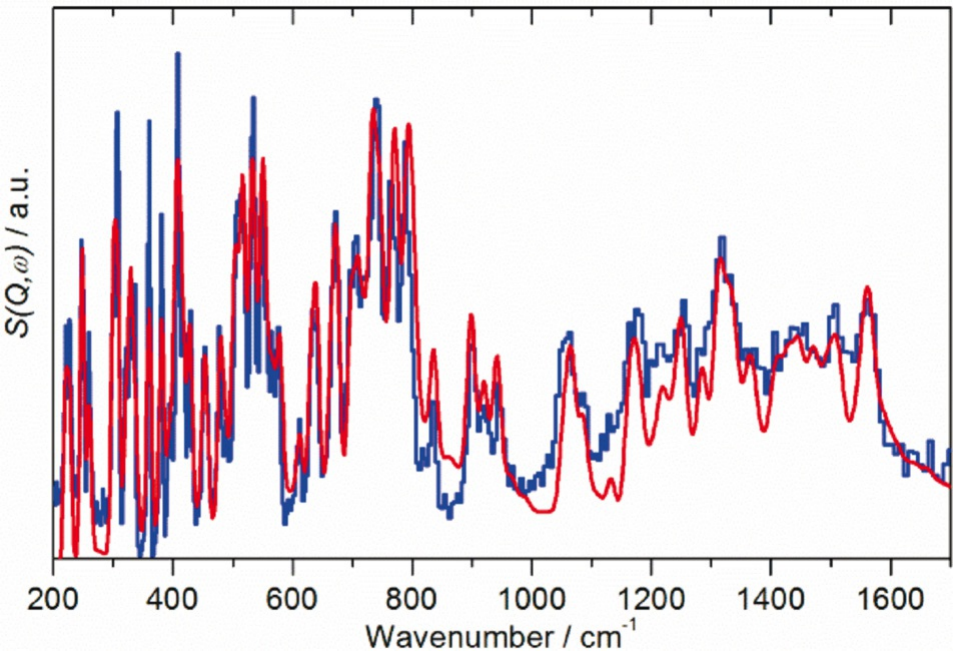
Comparison of the TOSCA INS spectrum of C_70_ at 7 K (blue) with that calculated for the solid state by CASTEP (red, site symmetry *C*_s_) after individual scaling of the modes and inclusion of the phonon wings.

Close inspection of Figure 5 a, b shows that the calculated and observed INS spectra differ in minor regards, in that some of the calculated transition energies are slightly (a few wavenumbers) inaccurate. We have previously shown that the calculated intensities (eigenvectors) are not very sensitive to the transition energy,^35^ so it is possible to make small changes in the transition energies and use the same eigenvectors. The procedure is to shift a calculated band to the nearest experimental feature of the correct intensity. In essence, this amounts to individual scaling factors for each of the modes and was successfully used for C_60_.^27^ Figure 8 shows the result, and it can be seen that the agreement is excellent; based on this, Table S2 in the Supporting Information lists the internal modes of C_70_ in *D*_5*h*_ symmetry. The transition energies reported are a combination of experimental and our ab initio results and represent the best available description of the vibrational modes of C_70_.

**Figure 9:**
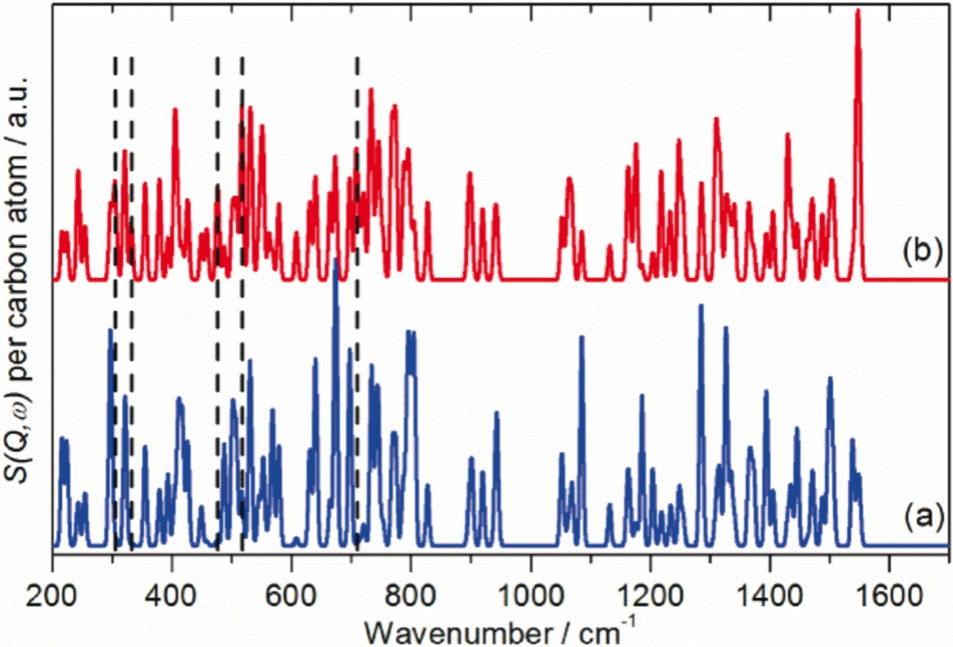
Calculated INS spectra of C_70_ modes involving motion of: a) the ‘belt’ atoms and b) the ‘cap’ atoms. The spectra are normalised to the intensity per carbon atom.

There have been attempts^19^,^26^ to identify modes that are characteristic of the ‘belt’ of the additional ten atoms that distinguish C_60_ from C_70_. By generating INS ‘spectra’ where the cross section of all the atoms except those of interest are set to zero, it is possible to see whether there are any modes localised in either the belt or the caps. Figure 9 shows the result, and it is clear that for almost all the modes, both the belt and cap atoms are involved. There are a few modes where there is no motion of the belt atoms (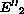
mode at 307 cm^−1^, 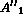
mode at 336 cm^−1^, 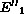
mode at 705 cm^−1^, 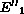
mode at 705 cm^−1^, and the 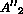
mode at 707 cm^−1^), and these are shown in Figure S1 in the Supporting Information. We find no modes that only involve motion of the belt atoms. We also disagree with the conclusion^19^ that the amplitude of motion of the belt atoms is larger than that of the cap: the intensities in Figure 9 are per carbon atom, and both spectra are plotted on the same ordinate scale. Thus the amplitudes of the atoms are similar in both cases.

## Conclusions

A combination of a much better quality INS spectrum with periodic DFT calculations has allowed all the internal modes of C_70_ to be assigned. Comparison of the INS spectra predicted by previous work with the new data demonstrates that all previous assignments were incorrect in several respects. In particular, isolated molecule calculations using Gaussian basis sets result in inaccurate assignments. In contrast, the periodic-DFT approach produces assignments that are in almost quantitative agreement with the data without the need for scaling for both C_60_ and C_70_. This clearly has implications for the future assignment of higher fullerenes such as C_84_.

The need for a reliable assignment spans astronomy,^5^,^6^ where the fullerenes are detected by their vibrational signatures, to polymers, where fullerenes are attracting attention as fillers^36^ or novel monomers^37^ and solar cells.^38^,^39^ There is considerable current interest in solar cells that use C_60_ or C_70_ derivatives as the light-harvesting element. Knowledge of the parent fullerene's vibrational spectrum is an essential first step in understanding how the spectrum is modified on derivatisation and how the materials change in use.

## Experimental Section

The INS^21^ experiments were performed with the high-resolution time-of-flight spectrometers, TOSCA^40^ and MAPS,^41^ at the ISIS^42^ pulsed spallation neutron source at the STFC Rutherford Appleton Laboratory, Chilton, UK. While both TOSCA and MAPS access the same energy transfer range, 0 to 4000 cm^−1^, they provide complementary data. In INS spectroscopy, overtones and combinations are allowed transitions, whose intensity depends on *Q*^2n^, where *Q* (Å^−1^) is the momentum transfer and *n* is the order of the transition, *n*=1 fundamental, *n*=2, first overtone or binary combination, and so forth. On the indirect geometry instrument, TOSCA, each energy-transfer value (*ω*, cm^−1^) is associated with a unique momentum-transfer value: *Q*^2^≈*ω*/16. Thus the intensity of the overtones and combinations increases rapidly with increasing energy transfer on TOSCA and dominate the spectrum at large energy transfer. The direct-geometry instrument, MAPS, can access low-momentum transfer at large-energy transfer, so it enables high energy modes to be observed that are difficult to detect on TOSCA. On TOSCA, the resolution is ∼1.25 % of the energy transfer across the entire energy range, while on MAPS, under the conditions used here, it is ∼1.5 % of the incident energy at the largest energy transfer and degrades with decreasing energy transfer. Thus, TOSCA provides excellent energy resolution at energy transfers<1200 cm^−1^; at larger energy transfer MAPS provides better resolution by virtue of the access to low *Q*. TOSCA and MAPS are highly complementary and enable the complete range of interest, 0–2000 cm^−1^, to be covered with good resolution. The spectra were recorded from a 9.1 g sample of C_70_ at 20 K.

Dispersive Raman spectra were recorded simultaneously with the TOSCA spectra with a modified Renishaw InVia spectrometer (Wotton-under-Edge, UK) using a diode laser at 785 nm as the excitation source and a Peltier-cooled CCD detector. The system has been described in detail elsewhere.^43^ The spectral resolution is determined by the laser wavelength and the dispersion of the grating and is ∼4 cm^−1^. The laser power at the sample is a few mW; this results in a 1 K temperature rise when the Raman spectrum is recorded. The Raman spectra have been corrected for the instrument response function. Weak sample fluorescence resulted in a sloping baseline; this has been approximated by a polynomial and subtracted from the spectrum.

Attenuated total internal reflection (ATR) infrared spectra (400–4000 cm^−1^) were recorded with a Bruker Vertex 70 spectrometer (Billerica, USA) (4 cm^−1^ resolution, 128 scans), with the sample held in a Specac Low-Temperature Golden Gate ATR (Orpington, UK) with a KBr beamsplitter and a deuterated triglycerine sulfate (DTGS) detector. The spectra were recorded over the temperature range 113–300 K. They exhibited a strongly sloping baseline; this was approximated by a polynomial and subtracted from the spectrum.

C_70_ (99+ %, MER Corporation, Tucson, USA) was dried overnight in a vacuum oven at 383 K before use. The weight loss was less than 0.1 %.

Ab initio DFT calculations of the isolated molecule in *D*_5*h*_ symmetry were carried out with GAUSSIAN 03.^44^ The rpbe1pbe functional with the 6–311g(d) basis set was used. Periodic-DFT studies of the crystalline structures were carried out using the plane-wave pseudopotential method implemented in the CASTEP code.^45^,^46^ Exchange and correlation were approximated using the PBE functional.^47^ A norm-conserving pseudopotential for carbon was generated using the kinetic-energy optimised method,^48^ with core radii of 1.2 *a*_0_ (s) and 1.54 *a*_0_ (p) in conjunction with a plane-wave cut-off energy of 750 eV. All the calculations were carried out at the Γ-point to reduce the computational cost. The equilibrium structure, an essential prerequisite for lattice dynamics calculations, was obtained by BFGS geometry optimization, after which the residual forces were converged to zero within 0.009 eV Å^−1^. Phonon frequencies were obtained by diagonalisation of dynamical matrices, computed using density-functional perturbation theory^46^ (DFPT). An analysis of the resulting eigenvectors was used to map the computed modes to the corresponding irreducible representations of the point group and assign IUPAC symmetry labels. DFPT was also used to compute the dielectric response and the Born effective charges, and from these, the mode oscillator strength tensor and infrared absorptivity were calculated. The Raman activity tensors were calculated using a hybrid finite displacement/DFPT method.^49^ The calculated infrared and Raman spectra were generated using the CASTEP utility ‘dos.pl’.^50^ The program ACLIMAX^51^ was used to produce the INS spectrum from the ab initio results. The INS spectra in Figure 4 b,c were generated by using the published assignments,^19^,^26^ but with the eigenvectors calculated in this work. As noted earlier, the eigenvectors are not very sensitive to the calculated eigenvalue,^35^ and the INS intensity per mode is almost constant (since the intensity does not vary greatly across the spectrum, and all modes are present). Only the Debye–Waller factor changes (since it depends on *Q*, and this is directly related to the energy transfer^21^,^40^), and this is included in the generation of the INS spectrum by ACLIMAX.

## Supporting Information

As a service to our authors and readers, this journal provides supporting information supplied by the authors. Such materials are peer reviewed and may be re-organized for online delivery, but are not copy-edited or typeset. Technical support issues arising from supporting information (other than missing files) should be addressed to the authors.
