# Supplementary material for: Assignment of the Internal Vibrational Modes of C70 by Inelastic Neutron Scattering Spectroscopy and Periodic-DFT
Source: ChemistryOpen. 2015 May 20;4(5):620–5. doi: 10.1002/open.201500069 (PMC4608530; doi:10.1002/open.201500069)
Supplement: Supplementary file 1 [file open0004-0620-sd1.pdf]

ChemistryOPEN

Supporting Information

© 2015 The Authors. Published by Wiley-VCH Verlag GmbH & Co. KGaA, Weinheim

Assignment of the Internal Vibrational Modes of C_{70} by Inelastic Neutron Scattering Spectroscopy and Periodic-DFT

Keith Refson[a, b] and Stewart F. Parker*[a]

open_201500069_sm_miscellaneous_information.pdf

Table S1: Correlation table for $D_{5h} \rightarrow C_{2v} \rightarrow C_s$

D_{5h}	C_{2v}	C_s
A_1'	A_1	A'
A_2'	B_1	A''
E_1'	$A_1 + B_1$	$2A'$
E_2'	$A_1 + B_1$	$2A'$
A_1''	A_2	A''
A_2''	B_2	A'
E_1''	$A_2 + B_2$	$2A''$
E_2''	$A_2 + B_2$	$2A''$

Table S2: Comparison of observed and calculated INS, Raman and infrared modes in C_{70}.
(s = strong, m = medium, w = weak, sh = shoulder, v = very)

Experimental			*Ab initio*			Assignment		
INS (7 K) / cm^{-1}	Raman (7 K) / cm^{-1}	Infrared (113 K) / cm^{-1}	CASTEP / cm^{-1}	Raman intensity / $Å^4$	Infrared intensity / km mol^{-1}	C_s	C_{2v}	D_{5h}
220			216	104.40	0.00	A'	B_1	E_2' (1)
226	233 vs		225	1334.83	1.40	A'	A_1	E_2' (1)
248			242	23.58	0.00	A''	B_2	E_1'' (1)
	254 m		245	26.86	0.00	A''	A_2	E_1'' (1)
259	268 vs		255	2040.90	0.44	A'	A_1	A_1' (1)
300	303 vw		297	79.11	1.27	A'	A_1	E_2' (2)
			299	104.09	0.77	A'	B_1	E_2' (2)
			303	0.31	0.00	A''	A_2	E_2'' (1)
307	311 vw		306	0.54	0.00	A''	B_2	E_2'' (1)
321	324 vw		320	3.52	0.12	A''	B_2	A_2'' (1)
330	329 vw		322	4.57	0.92	A'	B_1	E_1' (1)
			322	8.42	1.96	A'	A_1	E_1' (1)

336	337 vw		333	0.62	0.00	A''	A_2	A_1'' (1)
360	363 m		355	21.13	5.99	A'	A_1	E_1' (2)
			356	18.44	0.58	A'	B_1	E_1' (2)
381	377 vw		379	1.16	0.02	A''	B_2	E_2'' (2)
	384 vw		380	5.50	0.03	A''	A_2	E_2'' (2)
394			393	55.70	0.96	A'	A_1	A_1' (2)
408			404	1.17	0.02	A''	B_2	E_2'' (3)
			405	0.45	0.00	A''	A_2	E_2'' (3)
			407	6.92	0.00	A''	A_2	E_1'' (2)
			408	4.85	0.00	A''	B_2	E_1'' (2)
417	412 m		412	253.59	0.15	A'	A_1	E_1' (3)
	421 m		417	144.94	0.67	A'	B_1	E_1' (3)
430	436 w		425	12.43	0.03	A'	B_1	E_2' (3)
			427	131.73	0.06	A'	A_1	E_2' (3)
453	459 vs		449	2376.05	0.07	A'	A_1	A_1' (3)
		457 s	458	5.47	8.24	A''	B_2	A_2'' (2)

478			476	0.48	0.00	A''	A_2	E''_2 (4)
		483 w	477	1.31	0.00	A''	B_2	E''_2 (4)
489	482 w	492 w	487	288.78	0.64	A'	B_1	A'_2 (1)
508			501	42.47	0.20	A'	B_1	E'_2 (4)
	508 w	509 w	503	151.33	1.80	A'	A_1	E'_2 (4)
511		510 m	507	2.54	4.31	A'	A_1	E'_1 (4)
			508	4.40	1.22	A'	B_1	E'_1 (4)
516			515	0.71	0.01	A''	B_2	E''_2 (5)
	522 w	520 sh	517	1.01	0.01	A''	A_2	E''_2 (5)
			518	1.30	0.00	A''	A_2	E''_1 (3)
			519	1.70	0.06	A''	B_2	E''_1 (3)
534		532 s	529	13.48	106.88	A'	A_1	E'_1 (5)
			530	21.87	4.33	A'	B_1	E'_1 (5)
			531	2.94	7.59	A'	A_1	E'_2 (5)
	539 w		533	33.70	16.91	A'	A_2	A''_1 (2)
			534	0.79	0.06	A''	B_1	E'_2 (5)

			545	4.28	0.29	A'	B_1	A_2' (2)
548			550	7.11	0.00	A''	A_2	E_1'' (4)
		556 w	550	11.78	0.00	A''	B_2	E_1'' (4)
554			552	0.54	0.00	A''	B_2	E_2'' (6)
			554	1.22	0.01	A''	A_2	E_2'' (6)
566		566 w	563	0.23	16.67	A''	B_2	A_2'' (3)
	571 m		568	441.07	1.07	A'	A_1	A_1' (4)
576			578	4.74	18.56	A'	B_1	E_1' (6)
	580 w	576 w	580	99.74	14.88	A'	A_1	E_1' (6)
612			609	2.43	0.01	A''	A_2	A_1'' (3)
633		634 m	630	0.94	0.06	A''	A_2	E_2'' (7)
			632	0.29	0.00	A''	B_2	E_2'' (7)
		m	639	0.24	4.89	A'	B_1	A_2' (3)
639	645 w	642	641	45.89	2.04	A'	A_1	E_1' (7)
			641	12.23	2.50	A'	B_1	E_1' (7)
670	671 vw		664	1.11	0.08	A'	B_1	E_2' (6)

	671 vw	672 m	665	6.54	0.09	A'	A_1	E_2' (6)
			673	1.10	0.00	A''	A_1	E_1' (8)
673			673	1.76	0.00	A''	B_1	E_1' (8)
	677 w		673	4.29	38.17	A'	B_2	E_1'' (5)
			675	6.61	21.70	A'	A_2	E_1'' (5)
696		695 w	698	340.31	0.38	A'	B_1	E_2' (7)
			698	32.94	0.73	A'	A_1	E_2' (7)
	704 m		698	54.26	0.51	A'	A_1	A_1' (5)
706			707	5.27	0.11	A''	B_2	E_1'' (6)
			708	2.65	0.01	A''	A_2	E_1'' (6)
		707 w	710	3.93	0.15	A''	B_2	A_2'' (4)
	724 vw		715	25.88	2.23	A'	A_1	A_1' (6)
		727 w	720	6.01	0.00	A''	A_2	E_1'' (7)
			721	42.42	0.01	A''	B_2	E_1'' (7)
729			731	1.03	0.13	A''	A_2	E_2'' (8)
			731	2.85	0.00	A''	B_2	E_2'' (8)

736	741 m		734	518.16	0.02	A'	A_1	E_1' (9)
			734	380.13	0.55	A'	B_1	E_1' (9)
			734	50.31	0.02	A''	A_2	E_1'' (8)
			736	43.63	0.00	A''	B_2	E_1'' (8)
743			741	32.17	0.68	A'	B_1	E_2' (8)
	743 m		744	320.75	0.89	A'	A_1	E_2' (8)
			745	3.62	0.99	A'	B_1	A_2' (4)
748			747	62.53	0.07	A'	A_1	E_1' (10)
		748 m	748	16.41	19.10	A'	B_1	E_1' (10)
		762 w	753	1.02	0.04	A''	A_2	A_1'' (4)
764			767	0.49	0.01	A''	B_2	E_2'' (9)
			768	2.41	0.00	A''	A_2	E_2'' (9)
764			769	65.54	0.22	A'	A_1	E_2' (9)
	769 w		769	122.22	0.05	A'	B_1	E_2' (9)
772			774	6.21	0.00	A''	B_2	E_1'' (9)
			774	0.49	0.00	A''	A_2	E_1'' (9)

	775 w		774	323.92	0.30	A'	A_1	E_2' (10)
		774 vw	775	14.44	6.49	A'	B_1	E_2' (10)
			787	1.69	0.00	A''	A_2	A_1'' (5)
789			789	0.70	0.00	A''	A_2	E_2'' (10)
		786 sh	789	1.91	0.01	A''	B_2	E_2'' (10)
		795 w	793	88.75	6.52	A'	B_1	E_2' (11)
	787 w		794	474.05	1.53	A'	A_1	E_2' (11)
			796	3.47	0.00	A''	A_2	E_1'' (10)
			797	3.85	0.00	A''	B_2	E_1'' (10)
	792 vw		801	332.30	5.33	A'	B_1	A_2' (5)
			805	34.34	14.41	A'	A_1	E_1' (11)
818	797 w		806	315.71	27.07	A'	B_1	E_1' (11)
835			828	0.80	0.21	A'	A_1	E_2' (12)
			829	7.34	0.18	A'	B_1	E_2' (12)
900			896	3.94	0.04	A''	A_2	A_1'' (4)
		900 m	899	39.16	0.80	A'	B_1	E_1' (12)

			900	25.93	0.35	A'	A_1	E_1' (12)
			902	3.67	1.40	A''	B_2	A_2'' (6)
921		924 vw	919	0.24	0.03	A''	A_2	E_2'' (11)
			920	1.54	0.62	A''	B_2	E_2'' (11)
			940	10.53	0.05	A'	B_1	A_2' (6)
945	950 w	950 m	942	212.71	9.41	A'	A_1	E_2' (13)
			944	197.90	0.88	A'	B_1	E_2' (13)
1048			1051	8.32	0.00	A''	A_2	E_1'' (11)
			1053	16.32	0.00	A''	B_2	E_1'' (11)
	1064 s		1061	648.54	2.88	A'	A_1	A_1' (7)
1063			1065	65.54	0.34	A'	B_1	E_2' (14)
			1065	105.41	13.96	A'	A_1	E_2' (14)
			1069	1.43	0.00	A''	B_2	E_2'' (12)
			1070	0.60	0.12	A''	A_2	E_2'' (12)
1085			1085	20.58	30.63	A'	A_1	E_1' (13)
	1091 w	1090 s	1085	15.16	17.08	A'	B_1	E_1' (13)

		1135 w	1133	1.81	8.18	A''	B_2	A_2'' (6)
1164		1156 m	1161	1.73	0.04	A''	B_2	E_2'' (13)
			1162	1.53	0.01	A''	A_2	E_2'' (13)
			1165	2.48	0.05	A''	B_2	E_1'' (12)
			1166	4.95	0.03	A''	A_2	E_1'' (12)
1176		1183 m	1173	16.11	72.99	A'	B_1	E_1' (14)
			1176	123.93	173.19	A'	A_1	E_1' (14)
	1178 w		1177	632.00	4.46	A'	B_1	E_2' (15)
			1178	90.41	6.82	A'	A_1	E_2' (15)
	1190 s		1186	3778.02	3.39	A'	A_1	A_1' (8)
	1217w	1208 w	1204	788.70	2.54	A'	B_1	A_2' (7)
1213		1228 w	1217	2.74	1.38	A''	B_2	A_2'' (7)
			1218	92.32	0.02	A''	B_2	E_1'' (13)
1243			1220	47.22	0.12	A''	A_2	E_1'' (13)
	1231 s		1233	5968.09	16.60	A'	A_1	A_1' (9)
			1234	2.29	0.17	A''	A_2	A_1'' (7)

1255		1254 s	1246	49.32	10.65	A'	A_1	E_1' (15)
			1248	0.65	0.00	A''	B_2	E_2'' (14)
	1254 vw		1249	179.68	15.87	A'	B_1	E_1' (15)
			1250	0.69	0.01	A''	A_2	E_2'' (14)
	1260 w		1254	372.19	0.08	A'	B_1	E_2' (16)
			1256	95.01	6.16	A'	A_1	E_2' (16)
1294			1285	8.54	1.99	A'	B_1	E_1' (16)
			1286	107.45	0.01	A''	B_2	E_1'' (14)
	1299 w	1294 m	1286	435.24	2.47	A'	A_1	E_1' (16)
			1288	79.26	0.01	A''	A_2	E_1'' (14)
1319			1309	0.28	0.00	A''	B_2	E_2'' (15)
		1314 sh	1310	5.84	0.05	A''	A_2	E_2'' (15)
			1311	6.45	0.07	A''	B_2	E_1'' (15)
			1312	1.66	0.00	A''	A_2	E_1'' (15)
		1323 s	1313	2.14	31.72	A'	B_1	E_1' (17)
			1315	6.48	106.53	A'	A_1	E_1' (17)

			1317	0.71	0.00	A''	A_2	A_1'' (8)
			1318	2.93	0.09	A''	B_2	A_2'' (8)
1331			1327	44.01	7.64	A'	A_1	E_2' (17)
	1323 w		1327	570.94	0.72	A'	B_1	E_2' (17)
		1337 sh	1329	5.04	0.14	A'	B_1	A_2' (8)
			1332	2.65	0.01	A''	A_2	E_2'' (16)
			1335	3.69	1.24	A''	B_2	E_2'' (16)
		1353 w	1341	731.01	3.75	A'	A_1	E_2' (18)
	1327 w		1341	1583.95	2.68	A'	B_1	E_2' (18)
		1373 m	1365	97.69	24.90	A'	A_1	E_2' (19)
			1365	20.86	9.57	A'	B_1	E_2' (19)
			1369	25.01	0.00	A''	A_2	E_1'' (16)
		1384 w	1373	7.93	0.30	A''	B_2	E_1'' (16)
			1394	1.64	0.26	A''	A_2	E_2'' (17)
		1413 sh	1396	21.52	0.66	A''	B_2	E_2'' (17)
			1405	112.09	18.87	A'	A_1	E_1' (18)

			1406	178.73	21.45	A'	B_1	E_1' (18)
1439			1429	120.21	0.00	A''	B_2	E_1'' (17)
			1429	476.18	117.36	A'	A_1	E_1' (19)
	1437 m	1427 s	1431	507.61	133.81	A'	B_1	E_1' (19)
			1431	71.88	0.05	A''	A_2	E_1'' (17)
	1449 s		1436	4854.90	16.74	A'	A_1	A_1' (10)
			1439	194.44	1.81	A'	B_1	A_2' (9)
			1445	1.50	0.01	A''	A_2	E_2'' (18)
	1460 w		1447	16.75	0.18	A''	B_2	E_2'' (18)
		1462 m	1463	3.53	13.45	A''	B_2	A_2'' (9)
	1472 m		1468	1606.31	5.07	A'	A_1	A_1' (11)
			1472	171.59	10.31	A'	B_1	E_1' (20)
			1473	0.92	9.02	A'	A_1	E_1' (20)
			1487	780.26	1.44	A'	A_1	E_2' (20)
		1491 m	1488	38.16	0.23	A'	B_1	E_2' (20)
		1513 s	1498	414.78	0.02	A''	B_2	E_1'' (18)

1507			1501	47.93	0.05	A''	A_2	E_1'' (18)
			1502	6.75	0.43	A''	B_2	E_2'' (19)
			1503	21.48	0.11	A''	A_2	E_2'' (19)
	1515 m	1527 sh	1506	3257.24	6.86	A'	A_1	E_2' (21)
			1508	1098.06	3.47	A'	B_1	E_2' (21)
			1538	11.06	0.03	A''	A_2	E_2'' (20)
1563		1563 s	1543	26.76	208.39	A'	B_1	E_1' (21)
			1545	38.12	20.01	A'	A_1	E_1' (21)
			1547	67.12	0.02	A''	B_2	E_2'' (20)
			1547	36.88	2.73	A'	B_1	E_2' (22)
			1547	62.77	0.17	A''	A_1	E_1'' (19)
			1548	197.28	26.22	A'	A_2	E_2' (22)
			1548	42.49	0.02	A''	B_2	E_1'' (19)
			1549	96.06	0.07	A''	B_2	A_2'' (10)
			1550	145.66	0.06	A''	A_2	A_1'' (9)
	1566 s		1552	10922.06	40.53	A'	A_1	A_1' (12)

Table S3: Fundamental transition energies (cm^{-1}) of C_{70}.

A'_1	A'_2	E'_1	E'_2	A''_1	A''_2	E''_1	E''_2
259	489	330	220/227	336	321	248	307
394	548	360	300	539	455	414	381
451	639	417	426	612	566	520	408
571	752	511	508	762	707	548	478
704	792	534	534	795	911	680	520
724	945	576	671	900	1135	705	554
1064	1217	642	696	1247	1226	727	633
1190	1337	673	731	1330	1331	742	736
1231	1453	736	769	1566	1462	781	779
1449		748	775		1565	804	789
1472		818	800			1048	921
1566		900	835			1177	1085
		1085	945			1243	1164
		1183	1063			1299	1255
		1255	1189			1324	1319
		1294	1260			1384	1347
		1323	1331			1439	1413
		1420	1353			1513	1460
		1427	1373			1563	1517
		1487	1502				1553
		1563	1521				
			1563				

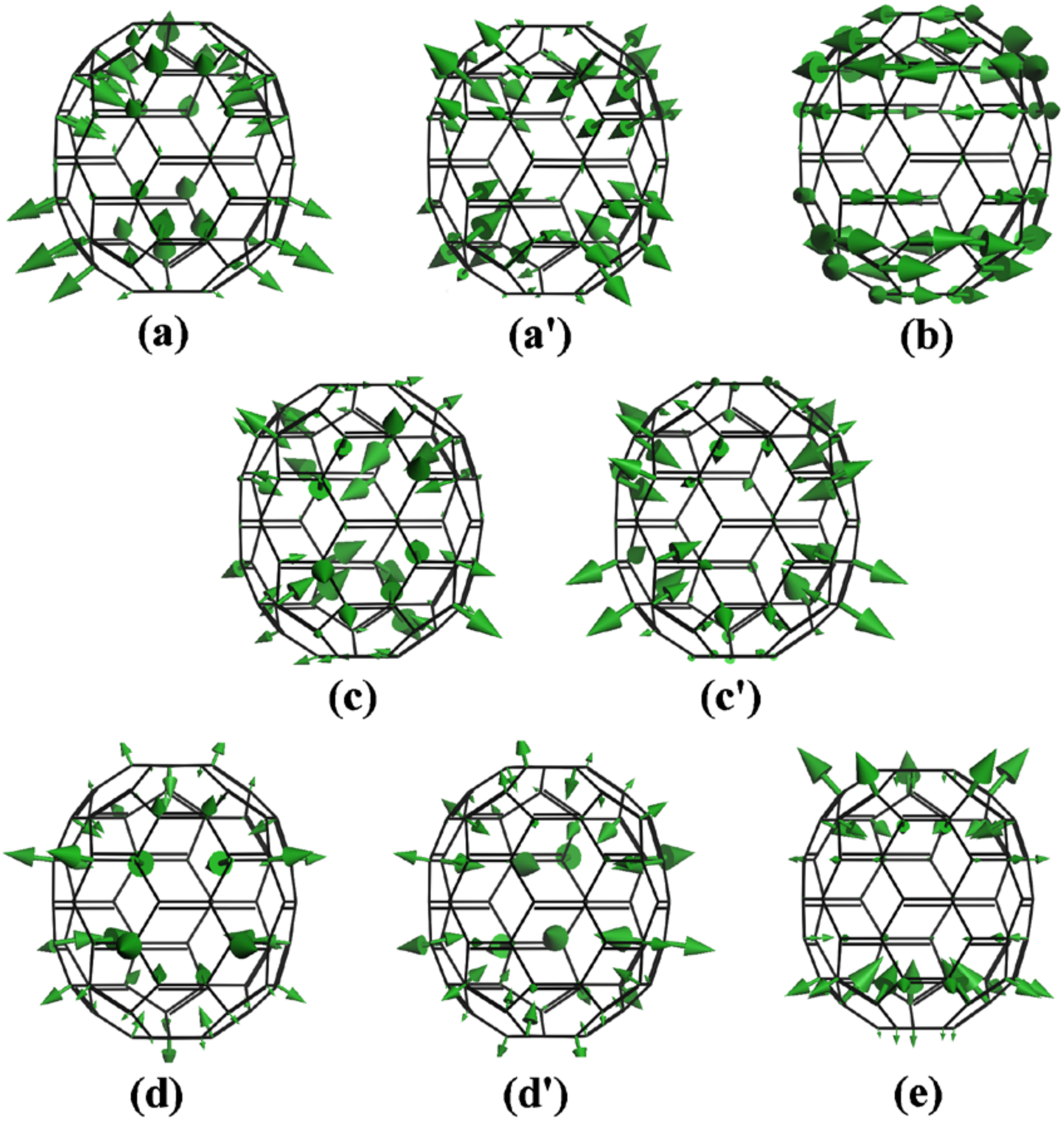

FIG. S1. Modes involving only the cap atoms of C_{70}. (a) and (a′) the two components of the E_2'' mode at 307 cm^{-1}, (b) the A_1'' mode at 336 cm^{-1}, (c) and (c′) the two components of the E_1'' mode at 705 cm^{-1}, (d) and (d′) the two components of the E_1'' mode at 705 cm^{-1} and (e) the A_2'' mode at 707 cm^{-1}.
